# Aldehyde dehydrogenase 1A1 confers intrinsic and acquired resistance to gemcitabine in human pancreatic adenocarcinoma MIA PaCa-2 cells

**DOI:** 10.3892/ijo.2012.1516

**Published:** 2012-06-12

**Authors:** HONG-QUAN DUONG, JAE SEOK HWANG, HEE JEONG KIM, HYO JIN KANG, YEON-SUN SEONG, INSOO BAE

**Affiliations:** 1 Departments of Oncology and; 2 Radiation Medicine, Lombardi Comprehensive Cancer Center, Georgetown University, Washington, DC, USA;; 3 World Class University (WCU) Research Center of Nanobiomedical Science, Dankook University, Cheonan;; 4 Department of Internal Medicine, Keimyung University College of Medicine, Daegu, Republic of Korea

**Keywords:** aldehyde dehydrogenase 1A1, gemcitabine resistance, apoptosis, human pancreatic adenocarcinoma, MIA PaCa-2 cells

## Abstract

Gemcitabine (GEM) is the front-line standard chemotherapy used for the treatment of pancreatic cancer; however, chemoresistance to GEM remains the major obstacle to the successful control of this disease. Both the expression levels and activity of aldehyde dehydrogenase 1A1 (ALDH1A1) are important features of tumor-initiating and/or cancer stem cell properties in multiple types of human cancer. As one of the intrinsic properties of cancer stem cells is drug resistance, in this study, we examined the correlation between the level and activity of endogenous ALDH1A1 and GEM resistance in the MIA PaCa-2 cell line that contains high expression levels and activity of ALDH1A1. We used small interfering RNAs (siRNAs) to deplete ALDH1A1 and investigate its potential role in conferring GEM resistance. The ALDH1A1 knockdown markedly reduced ALDH1A1 expression and activity and inhibited cell proliferation. Moreover, the combination of ALDH1A1-siRNA and GEM significantly decreased cell viability, increased apoptotic cell death and increased the accumulation of cells at the S-phase compared to the controls. Our data also demonstrated that ALDH1A1 expression and activity were significantly higher in the GEM-resistant MIA PaCa-2 cell line (MIA PaCa-2/GR), compared to the parental MIA PaCa-2 cell line (MIA PaCa-2/P). In the MIA PaCa-2/GR cells, the combination of ALDH1A1-siRNA and GEM also showed a significant decrease in cell viability and an increase in apoptotic cell death, emphasizing the importance of ALDH1A1 in both intrinsic and acquired GEM resistance. This potentially powerful combination treatment of ALDH1A1-siRNA and GEM warrants further investigation as an effective therapeutic regimen to overcome the resistance of pancreatic cancer to GEM.

## Introduction

Pancreatic cancer is the fourth most common cause of cancer-related mortality in the Western world. In the United States, approximately 36,800 people died from pancreatic cancer in 2010 (1). The overall prognosis for patients with pancreatic cancer remains poor: the five-year survival rate is less than 5% (2). A number of studies have evaluated various chemotherapeutic agents; however, only a few have produced results showing a significant improvement in survival (3).

Gemcitabine (GEM), a pyrimidine-based anti-metabolite, remains the first-line standard drug for the treatment of advanced human pancreatic cancer either alone or in combination with other chemotherapeutic agents (4–6). GEM is metabolized to gemcitabine triphosphate (dFdCTP) in cells and is incorporated into the DNA resulting in the induction of apoptosis due to the inhibition of DNA synthesis (7). However, the intrinsic and/or acquired resistance of pancreatic cancer to GEM presents a major challenge. Tumor cells acquire resistance to GEM by various mechanisms, including alterations in transport, drug targets and metabolism or in the genes regulating cell survival (8). The discovery of cancer stem cells (CSCs) as cancer-initiating components in leukemia and solid tumors has presented an attractive approach for treatment (9). Previous studies have shown that pancreatic CSCs are enriched in GEM-resistant cells (10,11). Most standard chemotherapy treatments destroy most of the tumor population; however, CSCs, which have intrinsic drug detoxifying and resistant mechanisms, can easily escape standard chemotherapy treatments.

Aldehyde dehydrogenase (ALDH) is a family of intra-cellular enzymes that participate in cellular detoxification, differentiation and drug resistance through the oxidation of cellular aldehydes (12). One of 17 ALDH isoforms, ALDH1A1 is a detoxifying enzyme responsible for oxidizing aldehydes to carboxylic acids and converting retinol to retinoic acid. It also holds the distinction of being a potential marker of CSCs and potentially playing a role in the biology of tumor-initiating cells (13–16). Tumor-initiating cell-enriched populations have been identified in multiple malignancies: breast, colon, pancreatic, lung, liver and ovarian cancer by using the AldeFluor assay, a functional flow cytometric assay that identifies cells with active ALDH1A1 (17–26).

In the present study, we performed experiments to determine whether the direct targeting of ALDH1A1 by small interfering RNA (siRNA) enhances the chemosensitivity of pancreatic cancer cells to GEM. Our results suggest that ALDH1A1 is a potentially important therapeutic target for human pancreatic ductal carcinoma cells.

## Materials and methods

### Cell culture and reagents

MIA PaCa-2, Panc-1, CFPAC-1 and BxPC-3 cells were purchased from the American Type Culture Collection (ATCC; Manassas, VA), and AsPC-1 and Colo-357 cells were obtained from the Tissue Culture Shared Resource of Georgetown University Lombardi Comprehensive Cancer Center (Washington, DC). The human pancreatic ductal epithelial cell line, HPDE6-C7, was acquired from Dr M.S. Tsao (27). AsPC-1, BxPC-3 and Colo-357 cells were cultured in RPMI-1640 medium supplemented with fetal bovine serum (FBS; 20% for AsPC-1, 10% for Colo-357 and BxPC-3 cells), 100 U/ml penicillin, 100 *μ*g/ml streptomycin and 1% sodium pyruvate. MIA PaCa-2 cells were cultured in Dulbecco’s modified Eagle’s medium (DMEM) containing 10% FBS, 2.5% horse serum (HS), 100 U/ml penicillin, and 100 *μ*g/ml streptomycin. Panc-1 and CFPAC-1 cells were cultured in DMEM containing 10% FBS, 10 U/ml penicillin, and 10 *μ*g/ml streptomycin. HPDE6-C7 cells were cultured in keratinocyte serum-free (KSF) medium supplemented by an epidermal growth factor and bovine pituitary extract and 1X antibiotic-antimycotic. Cell culture reagents were purchased from BioWhittaker (Walkersville, MD) and Invitrogen (Carlsbad, CA). GEM was obtained from Sigma (St. Louis, MO).

### Generation of GEM-resistant MIA PaCa-2 cells (MIA PaCa-2/GR cells)

The well-characterized pancreatic adenocarcinoma cell line, MIA PaCa-2 was used as the parental line (MIA PaCa-2/P) from which the GEM-resistant cell line was developed. The MIA PaCa-2/P cells were serially subcultured through incrementally increasing GEM concentrations, starting with 0.1 *μ*M for six months. MIA PaCa-2/GR cells retained the capacity for proliferation when returned to medium containing GEM (0.5 *μ*M).

### 3-(4,5-dimethylthiazol-2-yl)-2,5-diphenyltetrazolium bromide (MTT) assay

A total of either 2,000 MIA PaCa-2/P or MIA PaCa-2/GR cells were plated in 96-well flat bottom plates and then exposed to various concentrations of chemotherapeutic agents. At the indicated times, 10 *μ*l of 1 mg/ml MTT (Sigma) in phosphate-buffered saline (PBS) were added to each well for 4 h. After centrifugation and removal of the medium, 150 *μ*l of dimethylsulphoxide (DMSO) (Sigma) were added to each well to dissolve the formazan crystals. The absorbance was measured at 560 nm using an ELx808 Absorbance Microplate Reader (BioTek Instruments, Inc., Winooski, VT). The absorbance of untreated cells was designated at 100% and cell survival was expressed as a percentage of this value. Triplicate wells were assayed for each condition and standard deviation (SD) was determined.

### Western blot (WB) analysis

Cells were grown to ∼70% confluence and reagents were added at the indicated concentrations. After exposure to control-siRNA or ALDH1A1-siRNA with GEM, the cells were lysed in cell lysis buffer containing 20 mM Tris-HCl, 0.5 M NaCl, 0.25% Triton X-100, 1 mM EDTA, 1 mM EGTA, 10 mM β-glycerophosphate, 10 mM NaF, 300 *μ*M Na_3_VO_4_, 1 mM benzamidine, 2 *μ*M PMSF, and 1 mM DTT. The protein concentration was determined by a BCA protein assay kit (Thermo Scientific, Rockford, IL). Proteins were separated on SDS-PAGE, transferred onto a PVDF membrane, blocked in 1X blocking buffer (Sigma) and probed with the following primary antibodies: ALDH1A1 (Abcam, Cambridge, UK), poly(ADP-ribose) polymerase (PARP; BD Biosciences, Franklin, NJ), and α-tubulin (Sigma). The membranes were then incubated with horseradish peroxidase (HRP)-conjugated secondary antibodies (Sigma) and visualized with a chemiluminescence kit (Santa Cruz Biotechnology, Santa Cruz, CA) according to the manufacturer’s recommended protocol and exposed to X-ray film (American X-ray & Medical Supply, Jackson, CA).

### Flow cytometry

MIA PaCa-2 cells were collected after transfection and treatment with GEM by trypsinization, washed with PBS and fixed overnight in 70% ethanol at −20°C. The cells were then incubated with 20 *μ*g/ml propidium iodide and 40 *μ*g/ml RNase A in 1X PBS. The cells were analyzed on a FACSCalibur flow cytometer (Becton-Dickinson, San Jose, CA) at the Flow Cytometry and Cell Sorting Shared Resource, Lombardi Comprehensive Cancer Center, Georgetown University. The acquired data were analyzed by CellQuest Pro Analysis software (Becton-Dickinson).

### Caspase-3 activity assay

Caspase-3 activity assay was carried out by using a caspase-3/CPP32 colorimetric assay kit (BioVision, Mountain View, CA) according to the manufacturer’s instructions. MIA PaCa-2 cells transfected with siRNAs were treated with GEM for 48 h. Approximately 100 *μ*g of protein were incubated with 200 *μ*M Asp-Glu-Val-Asp-p-nitroanilide (DEVD-pNA) and 10 mM DTT at 37°C for 2 h. Absorbance was measured at 405 nm using an ELx808 Absorbance Microplate Reader (BioTekInstruments, Inc.). Increased CPP32 activity was determined by calculating these results according to the percentage of un-induced control samples.

### AldeFluor assay

Active ALDH1A1 was identified with the AldeFluor assay according to manufacturer’s instructions (StemCell Technologies, Durham, NC). The ALDH1A1-positive population was defined by cells with an increased FITC signal, with gates determined by diethylaminobenzaldehyde (DEAB)-treated cells (DEAB being an inhibitor of ALDH1A1 activity). The AldeFluor-positive cell population was measured by a FACSCalibur flow cytometer (Becton-Dickinson) and analyzed as described above.

### siRNAs

For the purpose of the RNA interference experiments, ALDH1A1-siRNA-1, 5′-GAACAGUGUGGGUGAAUUG-3′; ALDH1A1-siRNA-2, 5′-AGAGUACGGUUUCCAUGAA-3′; and control-siRNA, 5′-GACGAGCGGCACGUGCACA-3′, were purchased from Dharmacon Inc. (Lafayette, CO). The ALDH1A1-siRNA-1, ALDH1A1-siRNA-2 or control-siRNA were subsequently transfected into the MIA PaCa-2/P and MIA PaCa-2/GR cells using Lipofectamine™ 2000 (Invitrogen) according to the instructions of the manufacturer. The transfected cells were then processed for cell cycle analysis, WB analysis, caspase-3 activity, AldeFluor activity and cell proliferation.

### Trypan blue exclusion assay

Cells were collected after trypsinization of the cell monolayer, resuspended in serum-containing medium, stained with trypan blue and counted. Cell viability was evaluated via the trypan blue exclusion test using the Luna Cell Counter (Logos Biosystems, Gyunggi-do, Republic of Korea).

### Statistical methods

Statistical comparisons were made using the two-tailed Student’s t-test where appropriate. In all the experiments, the values, P<0.05, P<0.01 and P<0.001, were considered to indicate statistically significant differences. Data are expressed as the means ± SD.

## Results

### ALDH1A1 is differentially expressed in human pancreatic cancer cell lines and an immortal human pancreatic duct epithelial cell line

**Figure 1:**
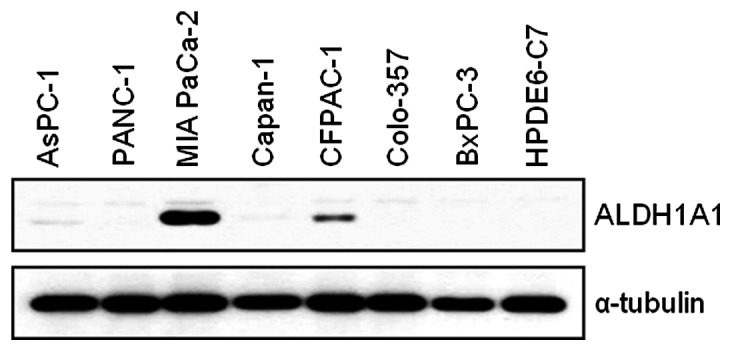
Basal expression level of aldehyde dehydrogenase 1A1 (ALDH1A1) in human pancreatic cancer cell lines (AsPC-1, Panc-1, MIA PaCa-2, Capan-1, CFPAC-1, Colo-357 and BxPC-3) and an immortal human pancreatic duct epithelial cell line (HPDE6-C7). Western blot analysis of exponentially growing cells was used to determine the expression level of ALDH1A1. Anti-α-tubulin antibody was used for loading and transfer control.

We first assessed the basal expression level of ALDH1A1 in AsPC-1, Panc-1, MIA PaCa-2, Capan-1, CFPAC-1, Colo-357, BxPC-3 and HPDE6-C7 cells. MIA PaCa-2 and CFPAC-1 cells expressed higher levels of ALDH1A1 than the other cell lines (Fig. 1). As we were interested in the potential contribution of ALDH1A1 to GEM-induced cytotoxicity, we performed further studies with ALDH1A1-positive MIA PaCa-2 cells.

### ALDH1A1 knockdown affects expression and activity of ALDH1A1 and cell proliferation

**Figure 2:**
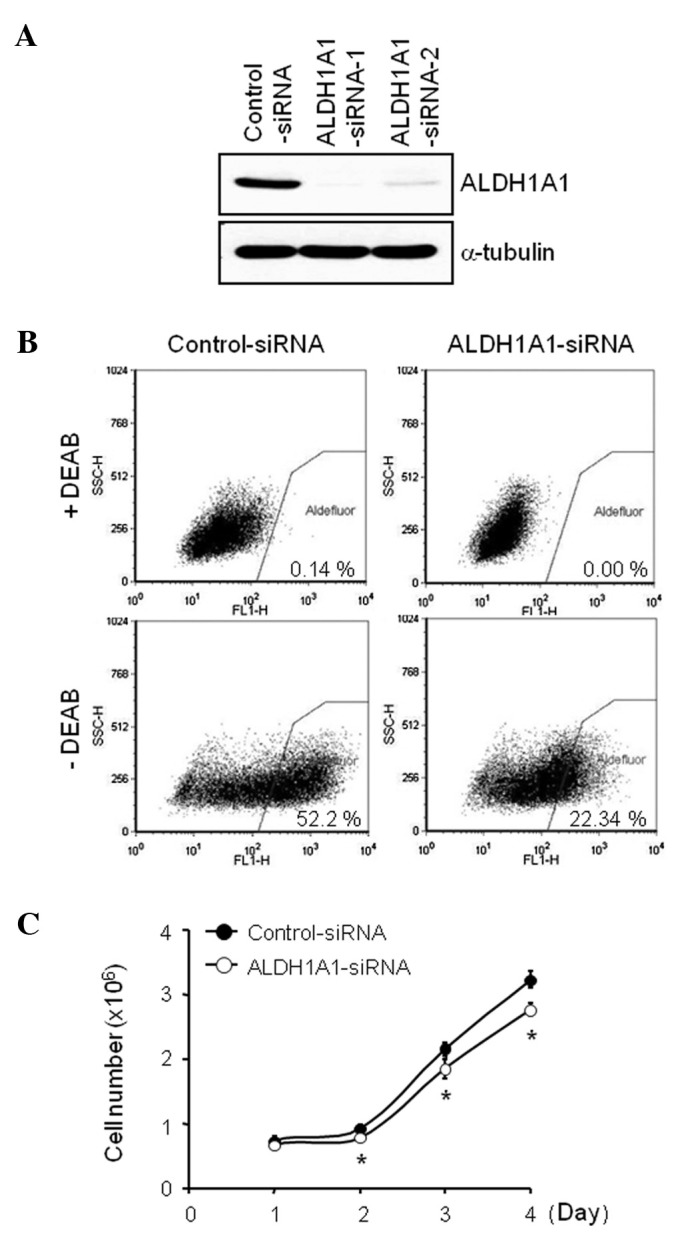
Aldehyde dehydrogenase 1A1 (ALDH1A1)-small interfering RNA (siRNA) inhibits ALDH1A1 expression and activity, and cell proliferation. (A) Western blot of MIA PaCa-2 cells transfected with ALDH1A1-specific-siRNA (vs. control-siRNA) for 72 h. Anti-ALDH1A1 antibody was used to measure the expression levels of ALDH1A1 and anti-α-tubulin antibody was used for loading and transfer control. (B) AldeFluor assay of MIA PaCa-2 cells transfected with ALDH1A1-siRNA or control-siRNA for 72 h. The ALDH1A1 inhibitor, diethylaminobenzaldehyde (DEAB), was added to ensure the accurate identification of ALDH1A1-positive and -negative cells. (C) Results from a trypan blue exclusion assay of MIA PaCa-2 cells, which were transfected with ALDH1A1-siRNA or control-siRNA for 1, 2, 3 and/or 4 days. Experiments were repeated three times and similar results were obtained. Error bars represent the standard deviation. ^*^P<0.05 represents significant difference between the ALDH1A1- and control-siRNA treated groups.

In order to investigate the significance of endogenous ALDH1A1 on GEM-induced cytotoxicity, we used the siRNA-based knockdown system. ALDH1A1-specific-siRNAs, targeting two different regions of ALDH1A1 sequences were designed and tested. Our transfection experiments with 100 nM of siRNA for 72 h demonstrated that ALDH1A1-siRNA-1 was more effective in reducing ALDH1A1 expression level than ALDH1A1-siRNA-2 (Fig. 2A). We then performed an AldeFluor assay, a functional flow cytometric assay that identifies cells with active ALDH1A1, to determine the effect of ALDH1A1-siRNA on ALDH1A1 activity. ALDH1A1 knockdown markedly reduced the AldeFluor-positive cell population from 52.2% by control-siRNA to 22.3% by ALDH1A1-siRNA at 72 h after administration (Fig. 2B). Moreover, the ALDH1A1 knockdown inhibited cell proliferation in a time-dependent manner (0, 1, 2, 3 and/or 4 days), compared to the control-siRNA (Fig. 2C). The overall number of attached cells was decreased by the ALDH1A1-siRNA knockdown (data not shown), and the loss of cell proliferation was observed from day one post-transfection (Fig. 2C).

### ALDH1A1 knockdown enhances cytotoxicity and apoptotic cell death induced by GEM

**Figure 3:**
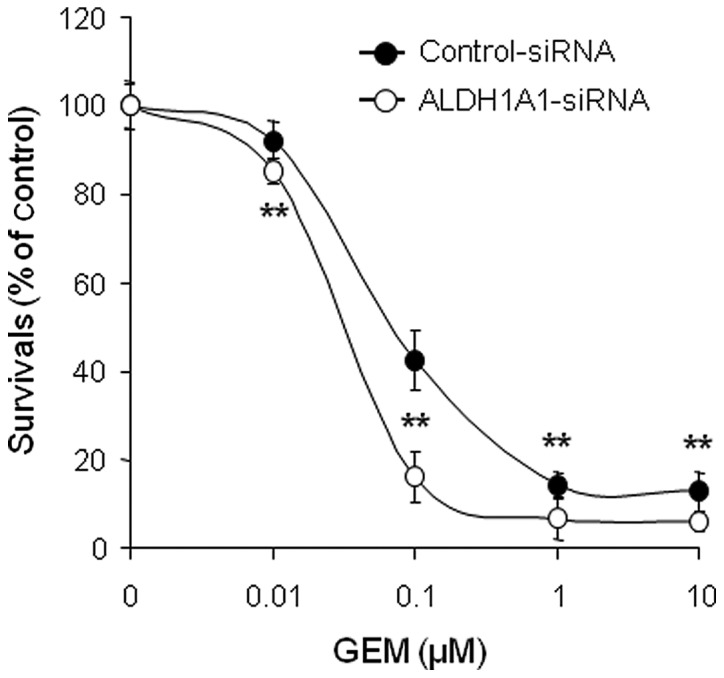
Knockdown of aldehyde dehydrogenase 1A1 (ALDH1A1) confers sensitivity to gemcitabine (GEM). An MTT assay of MIA PaCa-2 cells transfected with ALDH1A1-siRNA or control-siRNA for 48 h and further treated with GEM in a dose-dependent manner (0, 0.01, 0.1, 1 and/or 10 *μ*M) for 72 h was used to determine cell viability. Error bars represent standard deviation. ^**^P<0.01 represents significant difference between the ALDH1A1-siRNA plus GEM and control-siRNA plus GEM groups.

In order to investigate whether controlling ALDH1A1 expression levels can alter sensitivity to GEM, MIA PaCa-2 cells pre-treated with ALDH1A1- or control-siRNA for 48 h were incubated with various concentrations of GEM (0, 0.01, 0.1, 1 and/or 10 *μ*M) for 72 h and cell viability was determined by MTT assay. The results showed that the combination of ALDH1A1-siRNA plus GEM was significantly more effective at reducing survival than control-siRNA plus GEM (Fig. 3). To further evaluate the interaction between ALDH1A1-siRNA and GEM, we determined the half maximal inhibitory concentration (IC_50_) and found strong synergistic anti-tumor effects. The IC_50_ of GEM decreased from 0.16 *μ*M in the control-siRNA-treated cells to 0.035 *μ*M in the ALDH1A1-siRNA-treated cells.

**Figure 4:**
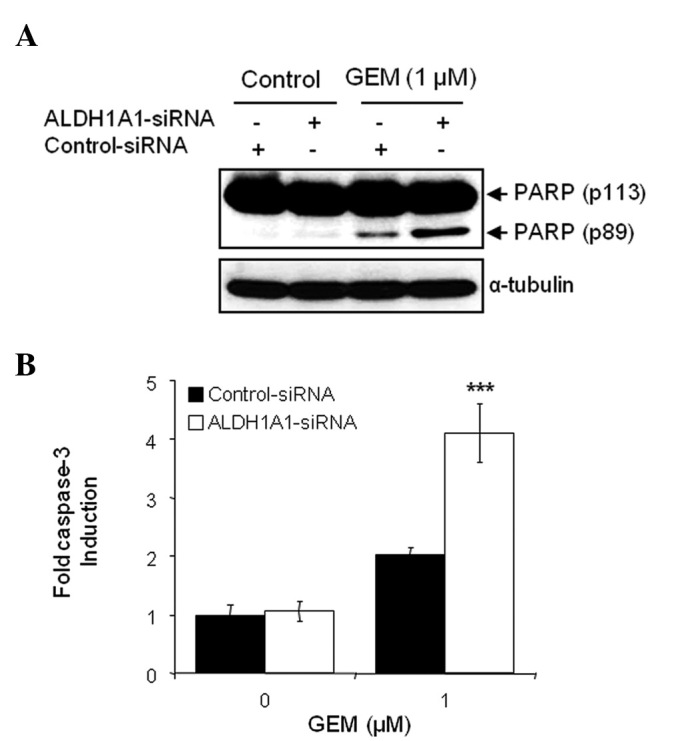
Aldehyde dehydrogenase 1A1 (ALDH1A1) knockdown enhances apoptotic cell death by gemcitabine (GEM). MIA PaCa-2 cells were transfected with ALDH1A1-siRNA or control-siRNA for 48 h, and then further treated with 1 *μ*M GEM for 48 h. (A) Western blot analysis for the detection of poly(ADP-ribose) polymerase (PARP) cleavage was used to measure apoptotic cell death. Anti-α-tubulin antibody was used for loading and transfer control. (B) A caspase-3 activity assay was also used to determine apoptotic cell death. Error bars represent the standard deviation. ^***^P<0.001 represents significant difference between the ALDH1A1-siRNA plus GEM and control-siRNA plus GEM group.

The combination of ALDH1A1-siRNA and GEM on the induction of apoptosis was also investigated. Cells pre-treated with siRNA for 48 h were exposed to 1 *μ*M GEM for 48 h. Apoptotic cell death was detected by WB analysis with the molecular biomarker of apoptosis, PARP cleavage. When comparing treatment with control-siRNA alone, control-siRNA plus GEM, ALDH1A1-siRNA alone or ALDH1A1-siRNA plus GEM, the latter treatment led to a more dramatic increase in cleaved PARP (Fig. 4A). We also performed a caspase-3 activity assay to confirm the effects of the ALDH1A1 knockdown on GEM-mediated apoptotic cell death. As expected, the combination of ALDH1A1-siRNA plus GEM most significantly increased caspase-3 activity (Fig. 4B).

### ALDH1A1 knockdown enhances induction of S-phase arrest by GEM

**Figure 5:**
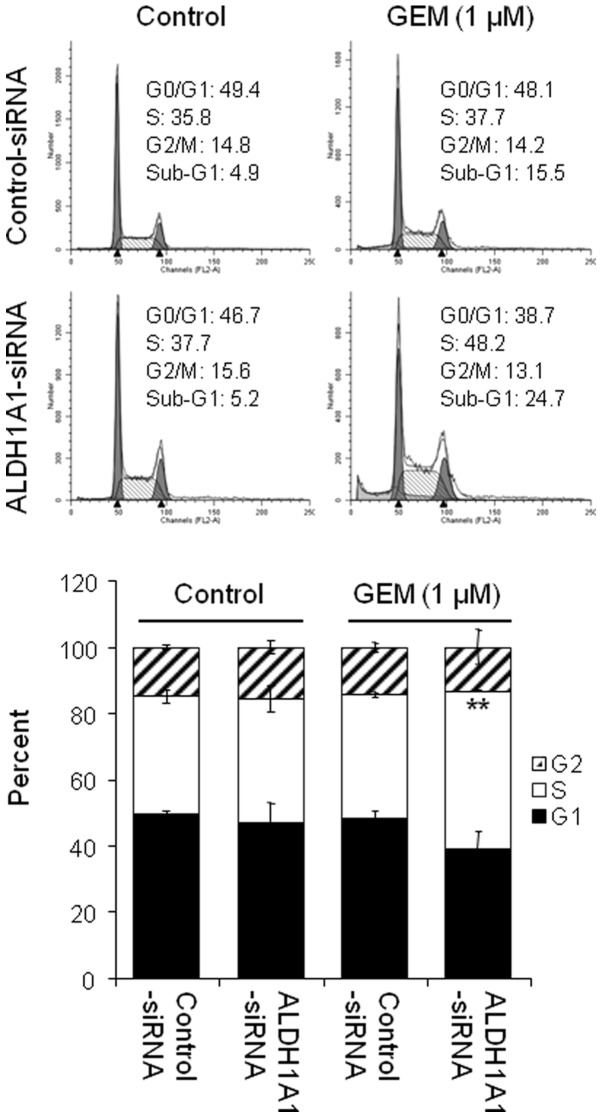
Aldehyde dehydrogenase 1A1 (ALDH1A1) knockdown enhances cell cycle arrest at the S-phase by gemcitabine GEM. Analysis by FACS of MIA PaCa-2 cells transfected with ALDH1A1-siRNA or control-siRNA for 48 h and then further treated with 1 *μ*M GEM for 48 h was used to determine cell cycle arrest. Experiments were repeated three times and similar results were obtained. Error bars represent the standard deviation. ^**^P<0.01 represents significant difference between the ALDH1A1-siRNA plus GEM and control-siRNA plus GEM group.

As GEM is known to accumulate in the S-phase of the cell cycle, we were interested in the effects of the ALDH1A1 knockdown on GEM-induced S-phase arrest. Cells pre-treated with ALDH1A1- or control-siRNA for 48 h were exposed to 1 *μ*M GEM for 48 h and their cell cycle profiles were assessed by FACS analysis. The data showed that the knockdown of ALDH1A1 or treatment with 1 *μ*M GEM alone induced a small increase in the accumulation of cells in the S-phase (from 35.8% by control-siRNA to 37.7% by ALDH1A1-siRNA or to 37.7% by 1 *μ*M GEM) (Fig. 5). However, the combined effects of ALDH1A1-siRNA plus GEM significantly increased the cell population in the S-phase (from 37.7% by control-siRNA plus GEM to 48.2% by ALDH1A1-siRNA plus GEM) (Fig. 5).

### ALDH1A1 knockdown enhances apoptotic cell death by GEM in MIA PaCa-2/GR cells

**Figure 6:**
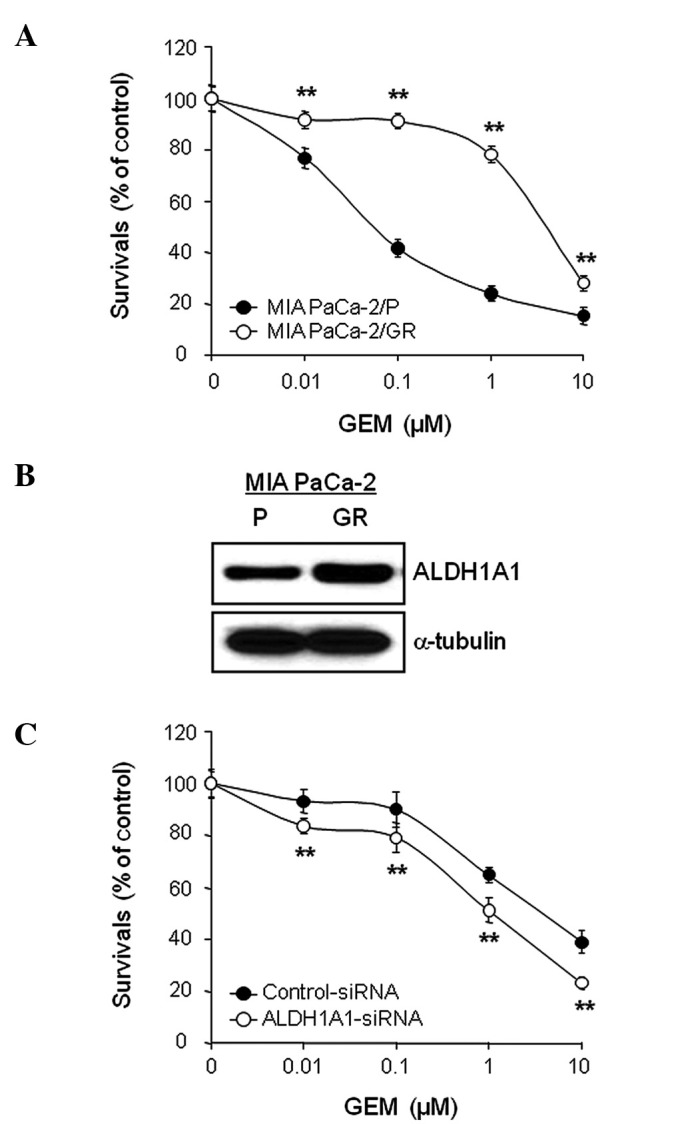
Aldehyde dehydrogenase 1A1 (ALDH1A1) knockdown enhances sensitivity of MIA PaCa-2/GR cells to GEM. (A) The results of MTT assay of MIA PaCa-2/P and MIA PaCa-2/GR cells, which were treated with gemcitabine (GEM) in a dose-dependent manner (0, 0.01, 0.1, 1 and/or 10 *μ*M) for 72 h. Error bars represent the standard deviation. ^**^P<0.01 represents significant difference between the MIA PaCa-2/GR cells plus GEM and MIA PaCa-2/P cells plus GEM groups. (B) Western blot analysis of exponentially growing MIA PaCa-2/P and MIA PaCa-2/GR cells was used to determine the expression level of ALDH1A1. (C) An MTT assay of MIA PaCa-2/GR cells transfected with ALDH1A1-siRNA or control-siRNA for 48 h, and then further treated with GEM in a dose-dependent manner (0, 0.01, 0.1, 1 and/or 10 *μ*M) for 72 h. Error bars represent the standard deviation. ^**^P<0.01 represents significant difference between the ALDH1A1-siRNA plus GEM and control-siRNA plus GEM groups.

MIA PaCa-2/GR cells were generated from MIA Paca-2/P cells that had been continuously exposed to increasing concentrations of GEM as described in Materials and methods. MIA PaCa-2/P and MIA PaCa-2/GR cells were treated with a various concentrations of GEM (0, 0.01, 0.1, 1 and/or 10 *μ*M) for 72 h and cell viability was evaluated by the MTT assay. The results showed that MIA PaCa-2/GR cells were relatively resistant to GEM with an IC_50_ value of 4.43 *μ*M, whereas MIA PaCa-2/P cells were relatively sensitive to GEM with an IC_50_ value of 0.11 *μ*M (Fig. 6A). The MIA PaCa-2/GR cells demonstrated a 40.3-fold higher resistant index towards GEM than MIA PaCa-2/P cells.

**Figure 7:**
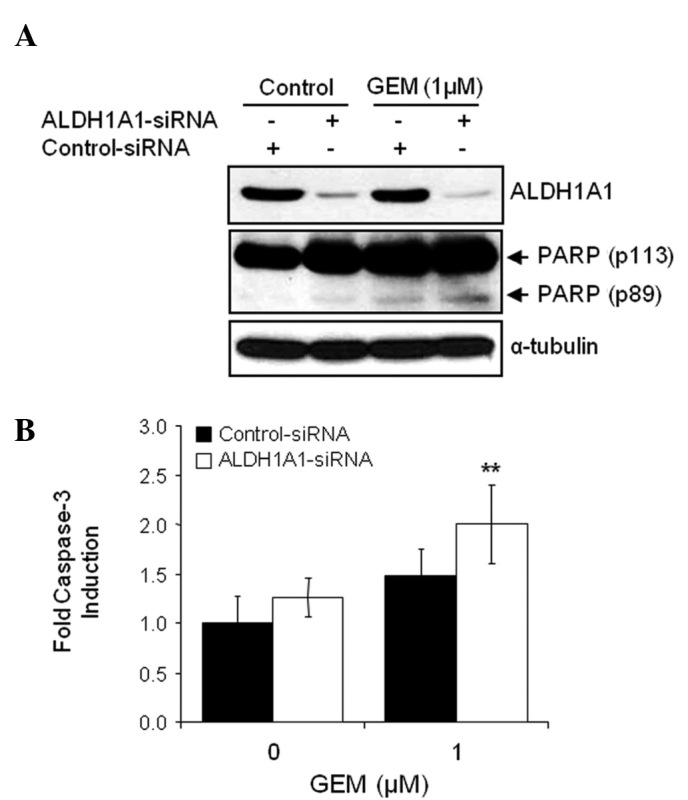
Aldehyde dehydrogenase 1A1 (ALDH1A1)-small interfering RNA (siRNA) increases apoptotic cell death by gemcitabine (GEM) in MIA PaCa-2/GR cells. MIA PaCa-2 cells were transfected with ALDH1A1-siRNA or control-siRNA for 48 h, and then further treated with 1 *μ*M GEM for 48 h. (A) Western blot analysis for the detection of poly(ADP-ribose) polymerase (PARP) cleavage was used to determine apoptotic cell death. (B) A caspase-3 activity assay for the detection of caspase-3 activity was also used to determine apoptotic cell death. Error bars represent the standard deviation. ^**^P<0.01 represents significant difference between the ALDH1A1-siRNA plus GEM and control-siRNA plus GEM group.

In order to compare the expression levels of ALDH1A1 between MIA PaCa-2/P and MIA PaCa-2/GR cells, cell lysates were obtained from exponentially growing cells and used for WB analysis. The expression level of ALDH1A1 was higher in MIA PaCa-2/GR cells than in the control MIA PaCa-2/P cells (Fig. 6B). Similarly, the AldeFluor-positive cell population was also higher in MIA PaCa-2/GR cells (data not shown). Finally, we investigated whether the increase in the ALDH1A1 expression level or activity in MIA PaCa-2/GR cells correlated with GEM resistance. MIA PaCa-2/GR cells pre-treated with ALDH1A1- or control-siRNA for 48 h were exposed to various concentrations of GEM (0, 0.01, 0.1, 1 and/or 10 *μ*M) for 72 h and cell viability was determined by MTT assay. The ALDH1A1-siRNA plus GEM combination demonstrated the most synergistic effect. The IC_50_ of GEM decreased from 4.54 *μ*M in the control-siRNA-treated cells to 0.94 *μ*M in the ALDH1A1-siRNA-treated cells (Fig. 6C). Moreover, we evaluated the effects of the ALDH1A1 knockdown on GEM-induced apoptosis in GEM-resistant cells. MIA PaCa-2/GR cells pre-treated with ALDH1A1- or control-siRNA for 48 h were exposed to 1 *μ*M GEM for 48 h. Treatment with ALDH1A1-siRNA plus GEM most significantly increased the level of cleaved PARP and caspase-3 activity in MIA PaCa-2/GR cells (Fig. 7A and B).

## Discussion

In this study, we investigated the significant role of ALDH1A1 in the chemoresistance of human pancreatic adenocarcinoma to GEM. We found that i) MIA PaCa-2 and CFPAC-1 cells express higher levels of ALDH1A1 than the other cell lines; ii) MIA PaCa-2/GR cells express higher levels of ALDH1A1 expression and activity than MIA PaCa-2/P cells; iii) the ALDH1A1 knockdown markedly reduced ALDH1A1 expression and activity and inhibited cell proliferation in MIA PaCa-2 cells; iv) the ALDH1A1 knockdown enhanced GEM-inhibited cell proliferation and GEM-induced apoptotic cell death not only in MIA PaCa-2/P cells, but also in MIA PaCa-2/GR cells and v) the ALDH1A1 knockdown enhanced GEM-induced S-phase arrest. These results strongly support the hypothesis that ALDH1A1 is an important determinant of chemoresistance to GEM. As far as we know, this is the first data showing that a combination of ALDH1A1-siRNA and GEM has synergistic anti-tumor effects in human pancreatic cancer cells.

ALDH1A1 expression characterizes a subpopulation of cells with tumor-initiating or CSC properties in several malignancies. ALDH1A1 expression has also been found in a subpopulation of cells with chemoresistance in numerous human cancer types. Based on genomic and proteomic profiles, a number of studies have shown that the levels of ALDH1A1 expression are significantly higher in platinum- or taxane-resistant ovarian cancer cells (26,28), classical and atypical multidrug-resistant gastric carcinoma cells (29), cyclophosphamide-resistant human carcinoma cells (30,31) and oxazaphosphorine-resistant human malignant blood cell lines (32). Moreover, ALDH1A1 has been proposed to play a significant role in the mechanism of resistance to cyclophosphamide in human carcinoma cells (30,31), oxazaphosphorine in human malignant blood cells (32) and platinum or taxane in human ovarian cancer cells (26). Shah *et al* and Hong *et al* showed that GEM-resistant cell lines had an increased expression of CD44, CD24 and ESA, which were reported as putative markers of pancreatic CSCs (10,11). These studies suggest that GEM preferentially targets more differentiated and rapidly proliferating pancreatic tumor cells, indicating the enrichment of the pancreatic CSC population in GEM-resistant pancreatic cancer cells. In the present study, we observed that the ALDH1A1-positive population in the GEM-resistant MIA PaCa-2 cells (MIA PaCa-2/GR) was enriched in the long-term treatment with GEM to establish resistant cell lines. Consistent with our results, Kallifatidis *et al* showed that long-term *in vitro* treatment with GEM for 21 days induced an enrichment of ALDH1A1-positive pancreatic CSCs (33). Taken together, these results suggest a promising strategy for targeting the pancreatic CSC population by targeting ALDH1A1 to contribute overcoming resistance to GEM.

Our study demonstrates that ALDH1A1 confers resistance to GEM in ALDH1A1-positive MIA PaCa-2 cells. ALDH1A1 is known to oxidize many intracellular aldehydes into carboxylic acids (34) and detoxify free oxygen radicals generated by chemotherapeutic agents. The induction of reactive oxygen species (ROS) has been described to increase mitochondrial membrane permeability and promote apoptosis. In a previous study, GEM markedly increased ROS production and the depletion of ROS significantly decreased GEM-induced growth suppression, indicating that ROS plays a role in GEM-mediated cytotoxicity in T3M4 pancreatic cancer cells (35). Thus, the high level of ALDH1A1 may reduce GEM cytotoxicity by efficiently detoxifying ROS generated by GEM. Moreover, either the ALDH1A1 knockdown or GEM treatment induced cell cycle arrest at the S-phase. In addition, the combined effects of ALDH1A1-siRNA plus GEM induced a greater accumulation of cells in the S-phase, which is critical for growth inhibition. Landen *et al* showed that the ALDH1A1 knockdown induced an accumulation of cells in the S- and G2-phase in taxane-resistant but not platinum-resistant ovarian cancer cells (26). However, the molecular mechanism of the ALDH1A1-siRNA-induced S-phase arrest is not clear at this point. Further studies are required to understand the function of ALDH1A1 in the regulation of the cell cycle.

In conclusion, in the present study, we demonstrate a potential significance of ALDH1A1 in two pancreatic cancer cell lines (MIA PaCa-2/P and MIA PaCa-2/GR). Reproducing these findings in other pancreatic cancer cell lines may help to determine whether the effects are cancer cell line-specific or not. Although ALDH1A1-positive cells were not isolated in this study, it may be useful to investigate the correlation between pancreatic CSCs and GEM resistance. Further studies on animal models will help to determine the significant role of ALDH1A1 in drug resistance.
